# Azithromycin reduces spontaneous and induced inflammation in ΔF508 cystic fibrosis mice

**DOI:** 10.1186/1465-9921-7-134

**Published:** 2006-10-25

**Authors:** Rachida Legssyer, François Huaux, Jean Lebacq, Monique Delos, Etienne Marbaix, Patrick Lebecque, Dominique Lison, Bob J Scholte, Pierre Wallemacq, Teresinha Leal

**Affiliations:** 1 Clinical Chemistry, Université Catholique de Louvain, Ave Hippocrate 10, Brussels, Belgium; 2 Industrial Toxicology and Occupational Medicine, Université Catholique de Louvain, Clos Chapelle aux Champs 30.54, Brussels, Belgium; 3 Cell Physiology, Université Catholique de Louvain, Ave Hippocrate 55, Brussels, Belgium; 4 Pathology, Louvain University Hospital at Mont-Godinne, Yvoir, Belgium; 5 Pathology, Université Catholique de Louvain, Ave Hippocrate 10, Brussels, Belgium; 6 Pneumology, Université Catholique de Louvain, Ave Hippocrate 10, Brussels, Belgium; 7 Erasmus University Medical Center, Cell Biology, Rotterdam, The Netherlands

## Abstract

**Background:**

Inflammation plays a critical role in lung disease development and progression in cystic fibrosis. Azithromycin is used for the treatment of cystic fibrosis lung disease, although its mechanisms of action are poorly understood. We tested the hypothesis that azithromycin modulates lung inflammation in cystic fibrosis mice.

**Methods:**

We monitored cellular and molecular inflammatory markers in lungs of cystic fibrosis mutant mice homozygous for the ΔF508 mutation and their littermate controls, either in baseline conditions or after induction of acute inflammation by intratracheal instillation of lipopolysaccharide from *Pseudomonas aeruginosa*, which would be independent of interactions of bacteria with epithelial cells. The effect of azithromycin pretreatment (10 mg/kg/day) given by oral administration for 4 weeks was evaluated.

**Results:**

In naive cystic fibrosis mice, a spontaneous lung inflammation was observed, characterized by macrophage and neutrophil infiltration, and increased intra-luminal content of the pro-inflammatory cytokine macrophage inflammatory protein-2. After induced inflammation, cystic fibrosis mice combined exaggerated cellular infiltration and lower anti-inflammatory interleukin-10 production. In cystic fibrosis mice, azithromycin attenuated cellular infiltration in both baseline and induced inflammatory condition, and inhibited cytokine (tumor necrosis factor-α and macrophage inflammatory protein-2) release in lipopolysaccharide-induced inflammation.

**Conclusion:**

Our findings further support the concept that inflammatory responses are upregulated in cystic fibrosis. Azithromycin reduces some lung inflammation outcome measures in cystic fibrosis mice. We postulate that some of the benefits of azithromycin treatment in cystic fibrosis patients are due to modulation of lung inflammation.

## Background

Cystic fibrosis (CF) is the most common, life-threatening, recessively inherited disease in Caucasian populations. CF results from mutations affecting a single gene encoding the CF transmembrane conductance regulator (CFTR) protein [1,2], which functions as a cyclic AMP-dependent low conductance chloride channel [3] but also has effects on the activity of other ion channels [4-6]. The most common CF mutation results in deletion of a single phenylalanine residue at position 508 (ΔF508) and causes defective synthesis and folding of the mutant protein that fails to transit from the endoplasmic reticulum and reach the apical membrane of many epithelial cell types [7]. CF has a complex phenotype with variable disease severity and multiple clinical manifestations including high concentrations of sweat electrolytes, exocrine pancreatic insufficiency, male infertility and sino-pulmonary disease [8]. The lung disease in CF is characterized by a self-sustaining cycle of airway obstruction, inflammation and infection. The high morbidity and mortality in CF is due to chronic respiratory infection, culminating in colonization with *Pseudomonas aeruginosa*, which has been implicated as an important stimulus in the progression of lung disease. This devastating complication, characterized by chronic unopposed neutrophil-dominated inflammation and progressive bronchiectasis, increases rates of lung function decline [9] and is a significant predictor of mortality [10].

Active treatment of lung disease is a cornerstone of CF management. This may include anti-inflammatory therapy approaches in combination with other conventional therapies such as antibiotics [11,12]. Azithromycin, a macrolide antibiotic structurally modified from erythromycin, has been used to treat CF patients resulting in significant clinical improvement in lung function with a reduction in pulmonary exacerbations and fewer courses of antibiotic use [13-16]. The precise mechanism of action of macrolides, apart from their antibactericidal actions [17-20], remains unclear. We hypothesized that azithromycin modulates lung inflammation in CF. We examined the effect of azithromycin pre-treatment on cellular and molecular parameters in the lungs of CF and normal homozygous wild-type mice with or without challenge with lipopolysaccharide from *Pseudomonas aeruginosa *(LPS), which would be independent of interactions of bacteria with epithelial cells [21-23].

## Methods

### Animal model

Young adult female CF mice homozygous for the ΔF508 mutation in the 129/FVB outbred background [24] and their wild-type littermates were housed in static isolator cages at the animal care specific pathogen free facility of the University of Louvain following recommendations of the Federation of European Laboratory Animal Science Associations (FELASA) [25]. In order to prevent intestinal obstruction CF mice were weaned to a liquid diet (Peptamen^®^, Nestlé Clinical Nutrition, France). Peptamen was replaced daily. Non-CF mice were fed with standard diet (Pavan Service-Carfil, Oud-Tournhout, Belgium) changed out once a week when cages were then sanitized and furnished with fresh bedding. Demineralized and acidified water was supplied *ad libitum*. The genotype of each animal was checked at 21 days of age using Taqman quantitative PCR multiplex analysis (Taqman, ABI PRISM^® ^7700 Sequence Detection System, Applied Biosystems, Foster, CA, USA) of tail clip DNA. Primers and Minor Groove Binder (MGB) probes designed for allele specific PCR using Primer Express Software (Applied Biosystems, Foster City, CA, USA) were as follows: forward primer = 5'-TTTCTTGGATTATGCCGGGTA-3'; reverse prime = 5'-GTTGGCAAGCTTTGACAACACT-3'; wild-type specific probe = 5'-FAM-AAACACCAAAGATGATATT-MGB-3'; mutant specific probe = 5'-VIC-AACACCAATAATATTTTC-MGB-3'. These studies and procedures were approved by the local Ethics Committee for Animal Welfare and conformed to the European Community regulations for animal use in research (CEE n° 86/609).

### Induction of lung inflammation

Sex and weight-matched CF and normal homozygous wild-type mice, 10 to 14 weeks of age, pre-treated with azithromycin (Pfizer, Brussels, Belgium) (10 mg/kg body weight/day, for 4 weeks, by oral administration using a pipette) and controls without azithromycin treatment were anesthetized with an i.p. mixture of 100 mg/kg ketamine (Parke-Davis, Ann Arbor, MI, USA) and 15 mg/kg xylazine (Bayer, Leverkusen, Germany). Acute lung inflammation was induced by instillation into the trachea through the mouth, using a laryngoscope and fine pipette tip, of 10 μg/20 g body weight of LPS (Sigma Chemical, St. Louis, MO, USA) in 50 μl saline [26]. Azithromycin treatment was stopped when LPS was administered.

### Bronchoalveolar lavage (BAL)

At selected time points after LPS instillation, mice were killed by i.p. injection of 20 mg sodium pentobarbital (Abbott, Chicago, IL, USA). BAL was performed by cannulating the trachea and lavaging with 1 ml sterile saline as described [27]. The BAL fluid (BALF) was centrifuged (250 × *g*, 10 min, 4°C) and the supernatant was aliquoted and stored at -20°C for further biochemical measurements. Differential cell counts were performed on cytospin preparations using DiffQuick staining (Dade, Brussels, Belgium).

### Biochemical analysis

#### Myeloperoxidase (MPO) activity

After BAL was performed, lungs were perfused via the right ventricle with saline and excised. MPO activity in lung homogenates was assessed at 490 nm over 10 min as previously described [28].

#### Lactate dehydrogenase (LDH) activity

LDH activity in BALF samples was assessed spectrophotometrically as described elsewhere [29].

#### Cytokine assays

Mouse macrophage inflammatory protein (MIP)-2, (R&D Systems, Minneapolis, MN, USA), tumor necrosis factor (TNF)-α and IL-10 (BD Pharmingen, San Diego, CA, USA) concentrations were measured in BALF using a standard sandwich enzyme-linked immunosorbent assay (ELISA) following the respective manufacturer's protocols. The detection limits of these ELISAs were respectively 1.5; 7.5 and 15.6 pg/ml. Biochemical analyses were performed in duplicate for each sample.

### Histopathology

Nonlavaged whole lungs were excised and inflation fixed via the trachea in 4% buffered paraformaldehyde and processed at 5 μm thickness for light microscopy. Slides were stained with hematoxilin and eosin or with Masson trichrome stain.

### Bacteriology

BALF samples were plated onto Columbia agar base with 5% sheep blood, a polyvalent non-selective medium. Sabouraud agar (Becton Dickinson, Franklin Lakes, NJ, USA) and Mac Conkey culture media were used to select for yeasts and fungi and for Gram negative bacteria, respectively. Plates were placed in a traditional incubator at 35°C for a minimum of 24 h. All tests were performed in duplicate.

### Statistics

Results are expressed as means ± SEM. Statistical data were analysed using SAS-JMP software (SAS Institute, Cary, NC, USA). Between-group comparisons were evaluated using one-way analysis of variance. Posthoc comparisons were made using Student's *t *test or Tukey-Kramer HSD test, as appropriate. Null hypothesis was rejected at p < 0.05. The alpha level was adjusted following Benferroni correction for pooled data from different experiments after identifying that means of normally distributed variables are not different (*t *test) and variances of populations are homogeneous (Snedecor's F test).

## Results

### Spontaneous lung inflammatory status in CF mice

**Figure 1:**
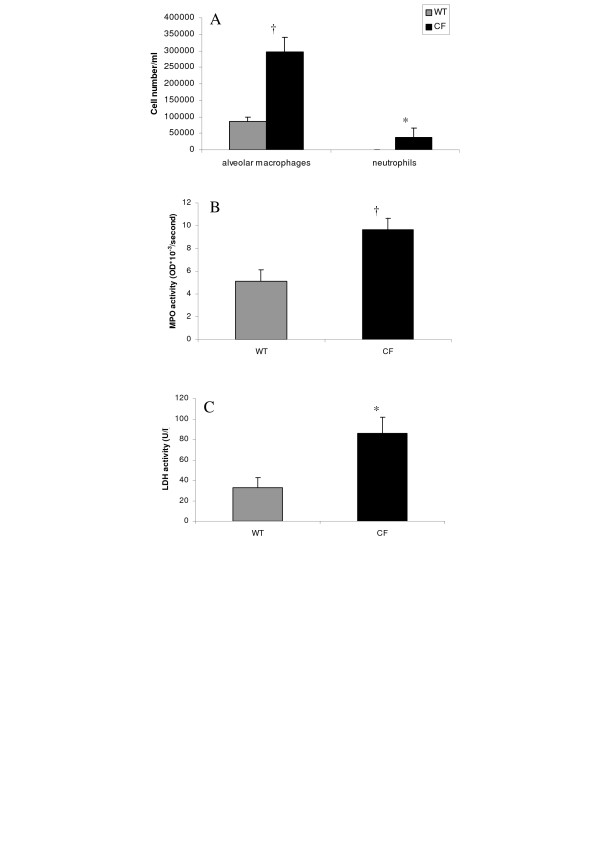
Cell composition of the bronchoalveolar lavage fluid (BALF) and tissue markers of lung inflammation from CF and wild-type (WT) mice under baseline conditions. **(A) **alveolar macrophages and neutrophil cell counts in BALF; **(B) **myeloperoxidase (MPO) activity in lung homogenates, expressed as optical density (OD) × 10^-3^/second; **(C) **lactate dehydrogenase (LDH) activity in BALF, expressed as U/L. Values are means ± SEM for 6–9 animals per group. *p < 0.05; †p < 0.01 for comparison of corresponding mean value *vs*. that obtained for the same parameter in the wild-type group of mice.

**Figure 2:**
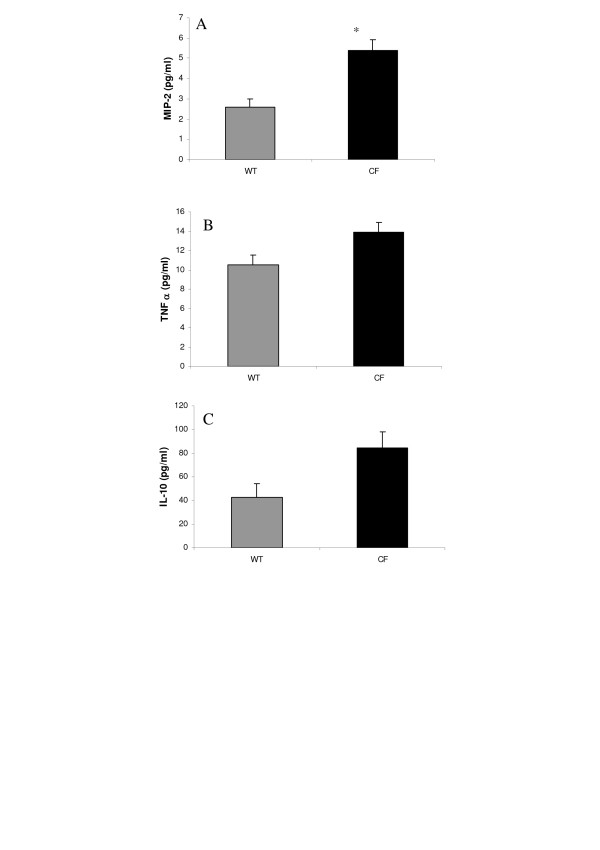
Inflammatory cytokine concentrations in airways from CF and wild-type (WT) mice under baseline conditions. **(A) **macrophage inflammatory protein-2 (MIP-2); **(B) **tumor necrosis factor-alpha (TNF-α); **(C) **interleukin (IL)-10. Values, expressed as pg/ml, are means ± SEM for 6–9 animals per group from the same experiment as that used to generate Figure 1. *p < 0.001 for comparison of corresponding mean value *vs*. that obtained in the wild-type group of mice.

Repeated bacteriological examination of BALF samples from CF and normal homozygous wild-type mice showed no known pathogenic infectious agents cultured in polyvalent media, and zero growth detected in selective culture media for yeast and fungus and for Gram negative bacteria (not shown). However, a spontaneous inflammatory status was observed in lungs of naive or not experimentally manipulated CF mice. Cellular profile in BALF samples from naive CF and wild-type mice showed striking differences in total and differential cell counts (Fig. 1A). As illustrated in Table 1 for animals not treated with azithromycin, the total number of cells was significantly higher in CF in comparison with wild-type mice. Macrophage counts were significantly higher in BALF samples from CF in comparison with wild-type mice (Fig. 1A, Table 1). Likewise, a spontaneous neutrophilia was observed in all of the 13 naive CF mice examined in two different experiments whereas it was always undetectable in naive wild-type mice (Table 1). MPO activity, an indicator of neutrophilic recruitment and activation status, was significantly increased in lung homogenates from naive CF mice (Fig. 1B). Moreover, LDH activity, an indicator of tissue injury, showed a significant increase in BALF samples from naive CF when compared to wild-type mice (Fig. 1C). MIP-2, a key chemokine in the recruitment of neutrophils functionally equivalent to the human IL-8, was about two-fold higher in naive CF than in wild-type mice. In the experiment illustrated in Fig. 2A, MIP-2 ranged from 2.9 to 6.5 pg/ml and from 1.5 to 3.5 pg/ml in naive CF and wild-type animals, respectively. Only one of the 9 CF animals showed a MIP-2 value within the range of those measured in control animals. No significant difference was seen between the two groups of animals with respect to concentrations of TNF-α (Fig. 2B; p: 0.21) and the anti-inflammatory cytokine IL-10 (Fig. 2C; p: 0.07).

**Table 1:** Cell composition of the bronchoalveolar lavage fluid and enzyme markers of lung inflammation from CF and wild-type (WT) mice in the absence of induction with *P aeruginosa *LPS, with and without pre-treatment with azithromycin (10 mg/kg/day), by oral administration, for 4 weeks.

		Alveolar Macrophages × 10^4^/ml	Neutrophils × 10^4^/ml	MPO OD × 10^-3^/second	LDH U/L
CF	AZM-	29.7 ± 4.2	2.8 ± 1.9	11.3 ± 1.2	108.7 ± 21.1
	AZM+	19.8 ± 1.5*	0.6 ± 0.4	7.7 ± 1.0*	91.8 ± 21.0
WT	AZM-	8.6 ± 1.4	0.0 ± 0.0	3.7 ± 0.7	67.6 ± 22.3
	AZM+	12.8 ± 1.5	0.0 ± 0.0	3.6 ± 0.4	70.6 ± 12.4

Values are means ± SEM for 9–13 animals per each of the 4 subgroups from pooled data obtained from two different sets of experiments. * p < 0.025 for comparison of corresponding mean value *vs*. that obtained for the same parameter in the same group of animal without pre-treatment with azithromycin.

**Figure 3:**
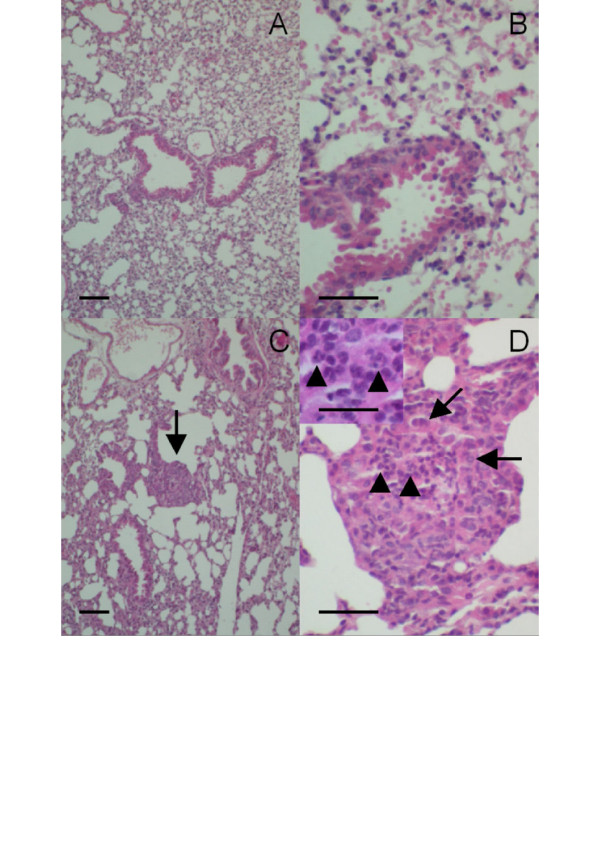
Hematoxylin and eosin-stained lung sections obtained from: (A-B) a naive wild-type mouse showing no pathological abnormalities; (C-D) a naive CF mouse showing focal areas of bronchopneumonia with neutrophil infiltration (arrow in C; focus enlarged at D). Arrowheads identify neutrophils in the bronchial lumen at D and in the inset; bronchial epithelial cells are identified by arrows. Calibration bars correspond to 50 μm in panels A-D and to 25 μm in the inset.

Histological findings confirmed the presence of spontaneous lung inflammation in naive CF mice. In contrast with age-matched wild-type littermates (Fig. 3A), focal areas of acute leukocytic bronchopneumonia were seen in lung sections from naive CF mice (Fig. 3B). Examination of Masson's trichrome sections showed no gross histologic evidence of increased collagen deposition in naive CF mice aged of 10–14 weeks when compared to wild-type animals (not shown).

### Exaggerated cell response to acutely LPS-induced inflammation in CF mice

**Figure 4:**
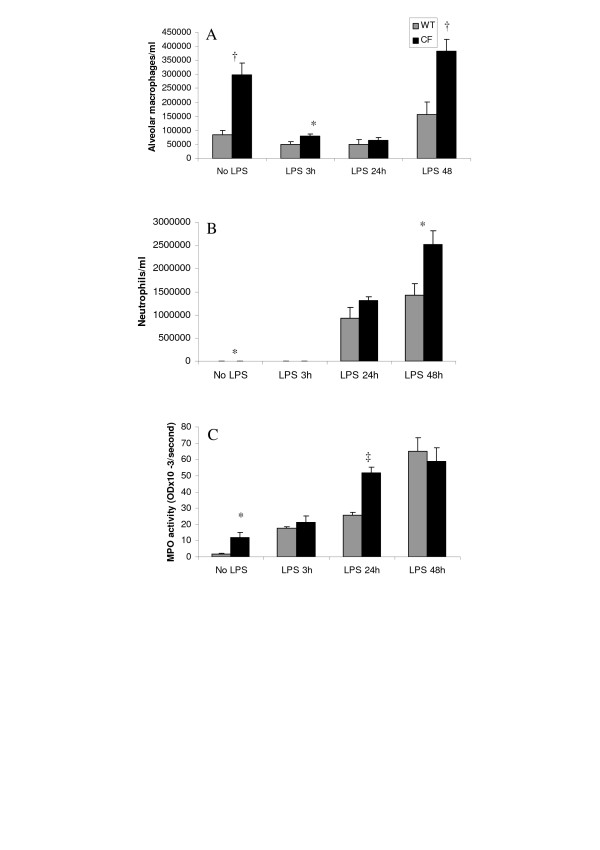
Time course of cell and tissue components of the inflammatory response in bronchoalveolar lavage fluid obtained 3 h, 24 h and 48 h after intratracheal instillation of *P aeruginosa *LPS from CF and wild-type (WT) mice. **(A) **alveolar macrophage counts; **(B) **neutrophil cell counts; **(C) **myeloperoxidase (MPO) activity in lung homogenates, expressed as OD × 10^-3^/second. Values are means ± SEM for 6–9 animals per each of the 8 subgroups. *p < 0.05; †p < 0.01; ‡p < 0.001 for comparison of corresponding mean value *vs*. that obtained at the same time point in the wild-type group of mice.

We next assessed the amplitude of pulmonary responses to LPS from *P aeruginosa *in CF and in wild-type mice. Intratracheal instillation of LPS was generally well tolerated by both mutant and normal animals, and no mortality was observed. A weight loss (up to 20%) was noted after 24 h and 48 h in LPS treated animals without any significant difference between wild-type and CF mice. After LPS exposure, a time-dependent cell infiltration was evident in both CF and wild-type mice. Macrophage numbers in BAL fluid were below control levels at 3 h and 24 h before coming back to baseline at 48 h postchallenge (Fig. 4A). The progressive and massive increase in neutrophil numbers was preceded by increase in MPO activity (Fig. 4B–C). Interestingly, the effect of LPS administration on cellular responses appeared to be different in CF compared to wild-type mice. At 48 h, the extent of both macrophage (Fig. 4A) and neutrophil count (Fig. 4B) was about two-fold higher in CF than in wild-type mice. The more exuberant neutrophil recruitment in CF mice was confirmed by monitoring the determination of MPO activity (Fig. 4C). In both groups of animals the enzyme activity continued to increase during 48 h being highest at the last time point evaluated when similar values were then noted in the two groups. However, at 24 h, a twice-higher level of MPO activity was reached in CF when compared to wild-type mice. No significant difference between the two groups of animals was seen on the LDH response after LPS treatment although a trend toward higher values was found at 24 h.

### Different LPS induced cytokine release patterns in CF and control mice

**Figure 5:**
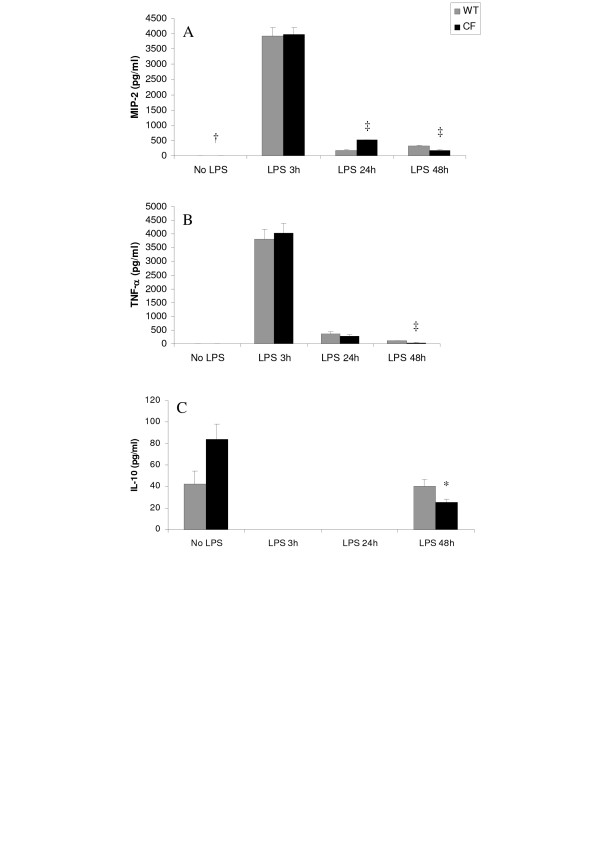
Time course of cytokine release in bronchoalveolar lavage fluid obtained 3 h, 24 h and 48 h after intratracheal instillation of *P aeruginosa *LPS from CF and wild-type (WT) mice. **(A) **macrophage inflammatory protein-2 (MIP-2); **(B) **tumor necrosis factor-alpha (TNF-α); **(C) **interleukin (IL)-10. Values, expressed as pg/ml, are means ± SEM for 6–9 animals per each of the 8 subgroups from the same experiment as that used to generate Figure 4. *p < 0.05; †p < 0.001; ‡p < 0.0005 for comparison of corresponding mean value *vs*. that obtained at the same time point in the wild-type group of mice.

Differences in the patterns of LPS-induced cytokine responses were revealed between the two groups of animals (Fig. 5A–C). A rapid release of MIP-2 (Fig. 5A) and TNF-α (Fig. 5B) was observed at 3 h after LPS instillation in wild-type and CF mice. At 24 h after LPS administration, when pro-inflammatory cytokine release responses progressively declined, MIP-2 levels remained significantly higher in CF than in wild-type mice (Fig. 5A). IL-10 production was not detectable at 3 h and 24 h after LPS challenge in both normal and mutant mice. However, at 48 h IL-10 was significantly reduced in CF animals compared to wild-type mice (Fig. 5C).

### Effect of azithromycin on the inflammatory response

To determine whether the use of azithromycin may reduce inflammatory responses in CF, we tested its activity in our animal model. Long-term (4 weeks), low dose (10 mg/kg body weight/day, by oral administration) azithromycin pre-treatment was performed in CF and wild-type mice either in baseline conditions or in LPS-induced inflammation. Azithromycin treatment reduced BALF macrophage numbers in CF mice treated as compared to those CF animals untreated with the macrolide (Table 1). MPO activity in lung homogenates and neutrophil infiltrate in BALF samples were also reduced, although the latter marker did not reach statistical significance. In contrast, in naive wild-type mice no effect of azithromycin was seen on cytotoxicity markers. Assessment of cytokine release did not show any noticeable effect of azithromycin in naïve CF mice (Table 2). In naive wild-type mice a marked increase of IL-10 level was noted after azithromycin treatment (Table 2).

**Table 2:** Inflammatory cytokine and chemokine concentrations in airways from CF and wild-type (WT) mice in the absence of induction with *P aeruginosa *LPS, with and without pre-treatment with azithromycin (10 mg/kg/day), by oral administration, for 4 weeks.

		MIP-2 pg/ml	TNF-α pg/ml	IL-10 pg/ml
CF	AZM-	4.9 ± 0.4	11.8 ± 1.8	84.2 ± 13.9
	AZM+	5.4 ± 0.7	9.3 ± 1.6	68.1 ± 7.9
WT	AZM-	2.9 ± 0.3	8.1 ± 1.6	42.3 ± 12.1
	AZM+	3.7 ± 0.4	10.6 ± 2.1	103.0 ± 19.1*

Values are means ± SEM for 9–13 animals per each of the 4 subgroups from pooled data obtained from the same sets of experiments as those used to generate Table 1. * p < 0.025 for comparison of corresponding mean value *vs*. that obtained for the same parameter in the same group of animal without pre-treatment with azithromycin.

Azithromycin treatment significantly reduced BALF cellularity in CF mice after LPS challenge (Table 3). At 48 h after LPS instillation, the degree of alveolar macrophage infiltrate was significantly decreased in CF mice pre-treated with azithromycin as compared to those CF animals without macrolide pre-treatment. At 24 h, the neutrophil count was reduced by half in azithromycin pre-treated CF mice as compared with the untreated CF group. In CF mice, pre-treatment with azithromycin significantly reduced MPO activity 48 h after LPS (Table 3) when levels similar to those observed in naive conditions were reached (Table 1). No significant effect on LDH activity was observed following azithromycin treatment in LPS treated CF mice although a trend toward lower values was observed at 24 h and 48 h after LPS (Table 3). No similar effect of azithromycin on cell recruitment or MPO activity was noted in wild-type mice at any time point after LPS instillation (Table 3).

**Table 3:** Cell composition of the bronchoalveolar lavage fluid and enzyme markers of lung inflammation from CF and wild-type (WT) mice at the indicated time points after a single dose of *P aeruginosa *LPS with and without pre-treatment with azithromycin (10 mg/kg/day), by oral administration, for 4 weeks.

			Alveolar Macrophages × 10^4^/ml	Neutrophils × 10^4^/ml	MPO OD × 10^-3^/second	LDH U/L
CF	LPS 3 h	AZM-	7.9 ± 0.6	0.6 ± 0.1	21.2 ± 4.2	50.0 ± 11.5
		AZM+	3.6 ± 0.7†	0.4 ± 0.2	30.1 ± 4.7	52.6 ± 17.6
	LPS 24 h	AZM-	6.4 ± 0.9	130.9 ± 7.9	51.7 ± 3.7	186.0 ± 61.2
		AZM+	6.4 ± 0.7	66.4 ± 10.3†	44.8 ± 3.3	98.2 ± 30.5
	LPS 48 h	AZM-	38.2 ± 4.3	232.9 ± 31.9	58.8 ± 8.2	155.6 ± 31.5
		AZM+	26.7 ± 2.4*	192.2 ± 27.0	15.4 ± 3.4‡	128.7 ± 21.8

WT	LPS 3 h	AZM-	5.0 ± 0.9	0.7 ± 0.1	17.7 ± 1.1	49.8 ± 20.6
		AZM+	4.4 ± 0.5	1.7 ± 0.9	21.4 ± 2.0	44.0 ± 6.7
	LPS 24 h	AZM-	5.0 ± 1.7	93.3 ± 23.3	25.8 ± 1.6	55.0 ± 22.3
		AZM+	7.1 ± 1.7	116.4 ± 22.7	31.2 ± 3.4	70.6 ± 18.6
	LPS 48 h	AZM-	15.7 ± 4.4	142.3 ± 25.7	64.9 ± 8.6	168.2 ± 25.3
		AZM+	14.1 ± 2.1	146.5 ± 17.0	54.9 ± 4.9	160.8 ± 19.1

Values are means ± SEM for 6–9 animals per each of the 12 subgroups. *p < 0.05; †p < 0.005; ‡p < 0.0005 for comparison of corresponding mean value *vs*. that obtained for the same parameter at the same time point without pre-treatment with azithromycin.

Significant effects of azithromycin treatment on LPS-induced cytokine release were noticeable in CF mice, notably on TNF-α at 3 h and 48 h (Table 4). At 24 h after LPS instillation, the MIP-2 response was reduced following pre-treatment with the macrolide (Table 4). Azithromycin did not modify the levels of IL-10 in CF mice 48 h after LPS (Table 4). In normal mice, TNF-α and MIP-2 responses were not modified after azithromycin pre-treatment in wild-type as they were in CF mice.

**Table 4:** Inflammatory cytokine concentrations in airways from CF and wild-type (WT) mice at the indicated time points after a single dose of *P aeruginosa *LPS with and without pre-treatment with azithromycin (10 mg/kg/day), by oral administration, for 4 weeks.

			MIP-2 pg/ml	TNF-α pg/ml	IL-10 pg/ml
CF	LPS 3 h	AZM-	3966.7 ± 230.3	4041.1 ± 347.6	ND
		AZM+	3800.8 ± 106.2	2648.1 ± 386.3*	ND
	LPS 24 h	AZM-	489.1 ± 20.4	282.3 ± 42.6	ND
		AZM+	300.4 ± 35.9†	251.9 ± 43.3	ND
	LPS 48 h	AZM-	166.9 ± 19.6	40.5 ± 9.7	25.2 ± 3.2
		AZM+	220.1 ± 26.9	14.2 ± 3.1*	30.2. ± 4.4

WT	LPS 3 h	AZM-	3933.3 ± 264.6	3805.2 ± 355.6	ND
		AZM+	4254.2 ± 142.0	4368.5 ± 362.5	ND
	LPS 24 h	AZM-	169.8 ± 35.3	368.9 ± 84.6	ND
		AZM+	230.0 ± 31.9	361.6 ± 29.9	ND
	LPS 48 h	AZM-	328.1 ± 13.5	114.9 ± 8.0	40.5 ± 6.4
		AZM+	340.0 ± 10.1	111.0 ± 11.1	36.7 ± 2.2

Values are means ± SEM for 6–9 animals per each of the 12 subgroups from the same experiment as that used to generate Table 3. *p < 0.05; †p < 0.005 and ‡p < 0.0005 for comparison of corresponding mean value *vs*. that obtained for the same parameter at the same time point without pre-treatment with azithromycin. ND = below corresponding detectable levels.

## Discussion

The present study was designed to explore the hypothesis that azithromycin modulates lung inflammation in CF. The growing interest in macrolide antibiotics as beneficial agents in CF followed the success of long-term erythromycin in the treatment of diffuse pan-bronchiolitis, a condition that exhibits striking similarities to CF [30-32]. It has been suggested that macrolides might exert their effect by normalizing ion transport across the CF respiratory epithelium [33]. However, it has been clearly demonstrated that the apparent beneficial effects of these drugs on pulmonary outcome in CF are not mediated by modulation of ion transport [34]. An open-label study [35] concluded that neither up-regulation of multi-drug resistance or CFTR proteins nor reduced bacterial adherence appear to be significant contributing mechanisms accounting for the beneficial results in clinical trials of macrolides in CF. We show that treatment with the macrolide in CF mice attenuated cellular infiltration in spontaneous and induced inflammatory conditions and inhibited pro-inflammatory cytokine release in LPS-induced inflammation.

We show that, in the absence of any detectable bacterial and fungal infection, young adult female CF mice generated in the 129/FVB background and homozygous for the most common CFTR gene mutation ΔF508 exhibit a spontaneous lung inflammatory status. The inflammation, confirmed histologically, is characterized by increased lung accumulation of macrophages and neutrophils, combined with upregulation of the pro-inflammatory cytokine MIP-2. This is associated with a higher degree of LDH activity, a biomarker of tissue damage. It has been reported that murine models of CF typically exhibit a range of severe and fatal intestinal pathologies but fail to develop significant CF like lung disease spontaneously [36-40]. However, Durie *et al *[41] have demonstrated age-related lung interstitial thickening and fibrosis in *cftr*-knockout mice. Spontaneous inflammation [42,43], excessive inflammatory responses and/or defective bacterial clearance after infection with *P aeruginosa *[44-49] have been reported in several CF mouse models. It has been postulated that the lack of spontaneous lung disease in most *Cftr*-knockout mouse strains, with the exception of a C57Bl/6 backcrossed strain, is related to the presence of an alternative (Calcium dependent) chloride channel function [42]. In agreement with the highly complex nature of CF lung pathology, phenotypic differences in CF mouse models have been observed relating to multiple factors such as the specific mutation, genetic background and environmental factors [45]. We show that while bacteriological studies have ruled out common bacteria and fungi, a possible involvement of a viral infection could not be excluded.

Irrespective of the presence of bacteria or other microorganisms, hypersusceptibility to environmental conditions between CF and wild-type mice could play a role in the spontaneous inflammatory status we found in naive CF mice. It was reported that knockout CF mice are more susceptible than normal homozygous and heterozygous mice to environmental *P aeruginosa *provided by non-sterile drinking water [49]. However, in our work, sterilised liquid diet was replaced daily; moreover, proper animal husbandry facilities including a self-contained microbiological unit with preventive measures and health monitoring were ensured following the FELASA recommendations for experimental units of laboratory animals established in our institution [25]. Possible nutritional differences, *i.e*. malnutrition, between *cftr*-knockout mice and wild-type mice were reported as being related to excessive inflammation in CF [50]. Nevertheless, van Heeckeren *et al *[51] found that dietary effects, such as Peptamen, are unlikely to account for differences in inflammatory responses to lung infection with mucoid *P aeruginosa*.

Our present data, showing spontaneous inflammation in naive CF mutant mice, appear to support clinical and experimental studies [52-55] suggesting that inflammation is a very early event and may occur even in the absence of bacterial pathogens. However, in a recent study it was claimed that in CF infants inflammation is a response to current or previous infection [56]. Whether CFTR dysfunction results directly in an increased predisposition to infection and whether inflammation arises independently from infection remains to be established. The characterization of a CF animal model expressing spontaneous lung disease and displaying exaggerated immune responses after induction of acute inflammation may represent an interesting preclinical model of disease and a useful *in vivo *system to assist in dissecting the pathogenesis of CF lung disease. The fact that our mouse model harbors a specific clinically relevant mutation represents an additional advantage for studying novel therapeutic strategies aiming at correcting intracellular trafficking and activation of the protein.

Spontaneous lung accumulation of neutrophils in our naive CF mutant mice is likely to be related to the elevated intra-luminal content of the chemo-attractant MIP-2. These data are in agreement with clinical [52-54,57,58] and experimental [55,59-61] studies suggesting that IL-8 levels are increased in CF. Blood neutrophils from patients with CF spontaneously secrete higher amounts of IL-8 [62]. Overall, neutrophil-derived toxic products may contribute to lung damage [63-65]. The degree of MPO activity in sputum has been positively correlated with airway injury and airflow obstruction in CF patients [66,67]. LDH activity, classically used to evaluate safety of agents analysed in different *in vitro *or *in vivo *experimental conditions [68], is increased in our naive CF mice indicating a higher index of cell injury.

The enhanced alveolar macrophages infiltrate observed in naive CF mice may play a major role in lung inflammation in CF. An activated status of alveolar macrophages may contribute to the triggering and development of inflammation in CF lung pathology. Accordingly, immunocytochemistry studies have demonstrated a higher percentage of CF than control alveolar macrophages expressing intracellular pro-inflammatory cytokines, IL-6 and IL-8 [59].

It has been proposed that CFTR is a cellular receptor for binding, endocytosing and clearing *P aeruginosa *from the normal lung [21-23]. In order to investigate inflammatory responses independently of alterations in clearance of the bacteria, we used, in the present work, intratracheal LPS as a challenge. As previously reported when using a model of LPS-induced lung inflammation in mice [69], macrophage numbers in BAL fluid were initially below control levels before coming back later on to baseline levels. Nevertheless, reasons why LPS treatment reduced early alveolar macrophage response are not clear. We could speculate that activation of pre-existing alveolar macrophages could result in increased cell adherence to the bronchoalveolar wall and cell attachment to the interstitial lung tissue making it more difficult to recover the cells during lavage procedure. Another possible indirect mechanism could be that during the early phase, some degree of bronchoconstriction secondary to LPS might contribute to technical difficulties in recovering alveolar macrophages during lavage. After LPS, effect on MPO activity preceded that on neutrophil count suggesting that LPS could initially act rather by increasing cell activation status. As previously described following aerosolisation of LPS from *P aeruginosa*, cellular and molecular responses were exaggerated in CF mice [70], suggesting that the latent inflammatory imbalance in CF may contribute to exert higher sensitivity to acute inflammatory responses [55]. Instillation of LPS into the trachea [26] allows delivery of accurate amounts of substance into the lungs. Indeed, by this method, smaller amounts of LPS, 1,000 lower than those previously described [70] were used in this work. It has been recently demonstrated in a selected CF mouse strain [47] that CFTR deficiency should be sufficient to produce increased inflammatory responses to a free-living mucoid *P aeruginosa *administered by insufflation into the lung. We show here more prominent release of pro-inflammatory cytokine MIP-2 in CF as compared with wild-type mice after acutely induced inflammation with LPS.

Downregulation of IL-10 may also be responsible for prolonged and excessive inflammatory responses in CF patients [59,71-73]. In our experiments we found reduced levels of IL-10 in the BALF of CF mice at 48 h after LPS challenge. In murine models relevant to CF lung disease, reduced levels of IL-10 have been associated with increased neutrophil infiltrate and increased activation of the transcriptional nuclear factor κB, NF-κB, in response to *P. aeruginosa *infection [74-76]. Exogenous IL-10 administration attenuated these excessive responses [76] suggesting that reduction of the anti-inflammatory cytokine expression may be responsible for prolonged and excessive inflammatory response in CF. Events involved in imbalance of inflammatory cytokines in CF lung disease are poorly understood and may include upregulation of NFκB [77], which modulates a large number of genes, particularly those involved in immune, inflammatory and anti-apoptotic responses [78,79].

Our data showed that in CF mutant animals, but not in normal animals, pre-treatment with azithromycin reduces the macrophage count in BALF before and after LPS challenge. It is well known that macrolides accumulate in the epithelial lining fluid of respiratory tract and easily enter the host defence cells, predominantly macrophages and neutrophils [80]. Direct correlations between neutrophil count and MPO activity seemed to be difficult to draw particularly while analysing combined time-dependent responses to LPS and azithromycin treatment when multiple pro-inflammatory and anti-inflammatory processes are playing simultaneously. Inhibition of neutrophil recruitment at 24 h after LPS in macrolide pre-treated CF mice could result, at least partly, from inhibition of neutrophil migration via reduction in pro-inflammatory cytokine MIP-2. A significant reduction in MPO activity was observed at 48 h after LPS exposure.

We observed a down-regulation of inflammatory cytokines (TNF-α and MIP-2) by azithromycin in CF mice. This may be related to results of a preliminary clinical trial in which reduction in sputum IL-8 levels was noted in 4 of the 6 patients treated with erythromycin [81]. In normal mice, azithromycin had no effect on cell infiltration and on pro-inflammatory cytokines, but increased IL-10 levels. Our results differ from those reported from an *in vivo *control mouse model of chronic endobronchial infection in response to mucoid *Pseudomonas *beads, in which macrolide treatment resulted in reduction of neutrophil infiltration and pro-inflammatory cytokines [82]. In contrast to our experimental conditions in which inflammation was investigated in the absence of infection, the effect of azithromycin could also be mediated by interactions with multiple virulence factors influencing the bacterial pathogenicity [82]. Accordingly, an *in vitro *study has suggested that the action of azithromycin could be mediated by interactions with the outer membrane of the bacteria [20]. Increased IL-10 levels following treatment with azithromycin in wild-type mice might contribute to an anti-inflammatory action of the macrolide. Yet, azithromycin did not increase IL-10 levels in CF mice. Although generally recognized as exerting a protective role, some evidence indicates that production of this pleiotropic cytokine can also lead to undesirable effects during inflammation and infection. Accordingly, administration of anti-IL-10 has been shown to enhance the survival in a murine model of *Klebsiella pneumoniae *infection [83]. Moreover, IL-10 production has been observed to be detrimental in lung function with *Streptococcus pneumoniae *in mice [84]. Since macrolide antibiotics exhibit their antimicrobial activities by interfering with the protein production of microorganisms, interference with protein production may be one mechanism by which cytokine release might be influenced by these drugs. Inhibition of cytokine production might also result from a modulation of gene expression. As downregulating effects of azithromycin are evident on pro-inflammatory cytokine release responses in CF mice, we could postulate that the macrolide exerts its action in CF lung disease on inhibiting TNF-α release and in less extent MIP-2, possibly by inhibiting NFκB pathway.

## Conclusion

Our results support the use of this CF mouse model as a valuable tool for studying human CF lung disease. This study further supports the concept that inflammation is a spontaneous abnormality in CF lung disease leading to exacerbation of immune responses when inflammation is acutely induced. Azithromycin reduces some lung inflammation outcome measures in CF mice. We postulate that some of the benefits of azithromycin treatment in CF patients is due to modulating lung inflammation probably by reducing, at least partly, cellular infiltration and pro-inflammatory cytokine expression.

## Competing interests

The author(s) declare that they have no competing interests.

## Authors' contributions

RL performed animal studies and biochemical analyses and helped drafting the manuscript.

FH contributed to the study design, the evaluation of data and the preparation of the manuscript.

TL designed and coordinated the study.

MD, EM performed pathological studies.

PL, JL, PW contributed to the study design.

DL, BJS revised the manuscript.

All authors have read the manuscript and approved the final manuscript.
